# Enhanced fingerprint classification through modified PCA with SVD and invariant moments

**DOI:** 10.3389/frai.2024.1433494

**Published:** 2024-08-05

**Authors:** Ala Balti, Abdelaziz Hamdi, Sabeur Abid, Mohamed Moncef Ben Khelifa, Mounir Sayadi

**Affiliations:** ^1^Research Laboratory SIME, ENSIT, University of Tunis, Tunis, Tunisia; ^2^J-AP2S Laboratory, South University, Toulon, France; ^3^NOCCS Research Laboratory, ENISo, ISITCOM, University of Sousse, Sousse, Tunisia

**Keywords:** fingerprint classification, fingerprint features, invariant moments, PCA, recognition, SVD features

## Abstract

This research introduces a novel MOMENTS-SVD vector for fingerprint identification, combining invariant moments and SVD (Singular Value Decomposition), enhanced by a modified PCA (Principal Component Analysis). Our method extracts unique fingerprint features using SVD and invariant moments, followed by classification with Euclidean distance and neural networks. The MOMENTS-SVD vector reduces computational complexity by outperforming current models. Using the Equal Error Rate (EER) and ROC curve, a comparative study across databases (CASIA V5, FVC 2002, 2004, 2006) assesses our method against ResNet, VGG19, Neuro Fuzzy, DCT Features, and Invariant Moments, proving enhanced accuracy and robustness.

## 1 Introduction

Fingerprint is a key component of biometric identification, several systems highlight singularity features such as core and delta point detection. Balti introduced a fingerprint verification system utilizing a backpropagation neural network (Balti et al., 2013), although singularity-based methods faced challenges, particularly their sensitivity to noise due to the local nature of singularities. Fitz explored Fourier transform features for classification, but the global features extracted by the wedge-ring detector lacked discriminatory information (Fitz and Green, 1996). In addition, Singular Value Decomposition (SVD) was examined by Balti et al. (2012), Balti and Sayadi (2014), Balti et al. (2014) as a potential tool for fingerprint characterization and identification. Their work focuses on the efficient extraction of significant features from fingerprint images through dimensionality reduction, as well as the identification of underlying fingerprint patterns through SVD. The goal of this approach is to obtain the most important data for precise fingerprint identification (Balti and Sayadi, 2014). In 2012, they investigated the potential advantages of integrating invariant moment features, which are immune to image changes, with SVD. This combined method may increase fingerprint characterization accuracy.

In recent years, Convolutional Neural Networks (CNN) have been widely used, even in the field of fingerprint recognition. For instance, when I searched the literature, I found several significant studies in this area. These are some prominent instances, Militello et al. (2021) study to evaluate the efficacy of pre-trained convolutional neural networks (CNN) for fingerprint classification. Their study demonstrates how important classification is to reduce the quantity of comparisons required for large fingerprint databases. They tested the AlexNet, GoogLeNet, and ResNet architectures. They are noteworthy for being the first to thoroughly compare these popular CNN architectures for fingerprint classification.

By integrating multi-augmentation and inversion techniques into convolutional neural networks, Garg et al. (2024) have presented a novel approach to fingerprint recognition. They get around the issue of having little training data by creating new fingerprint images for each feature map using a variety of augmentation techniques and inversion. Using multiple CNN, the suggested method by Garg et al. (2024) extracts features from the augmented data. Significant improvements in accuracy were observed in their experiments with pre-trained models, such as VGG19. On the FVC2000_DB4 dataset, the VGG19 model outperformed other models with an accuracy of 97% thanks to multi-augmentation.

On the other hand, I discovered that researchers (Srivastava et al., 2022) have proposed a multimodal biometric framework for human identity validation using iris and finger-knuckle print (FKP) recognition. By combining multiple biometric traits (FKP and iris in this case), their method achieves higher accuracy. The framework extracts features from FKP images using SIFT and SURF, and extracts features from iris images using Log Gabor wavelets with PCA. Remarkably, they achieve 98.68% accuracy on the CASIA databases using a neuro-fuzzy classifier for match score level fusion.

To address these challenges, our work adopts Singular Value Decomposition (SVD) (Golshan and Mohammadi, 2013) and invariant moments (Hu, 1962) for fingerprint image verification. SVD, a well-established technique in digital image processing, efficiently reduces data volume, preserving essential features in a compact representation. Invariant moments, an effective image processing method, provide a thorough overview of texture with seven moments that are unaffected by translation, rotation, and adjustments to scale.

**Figure 1:**
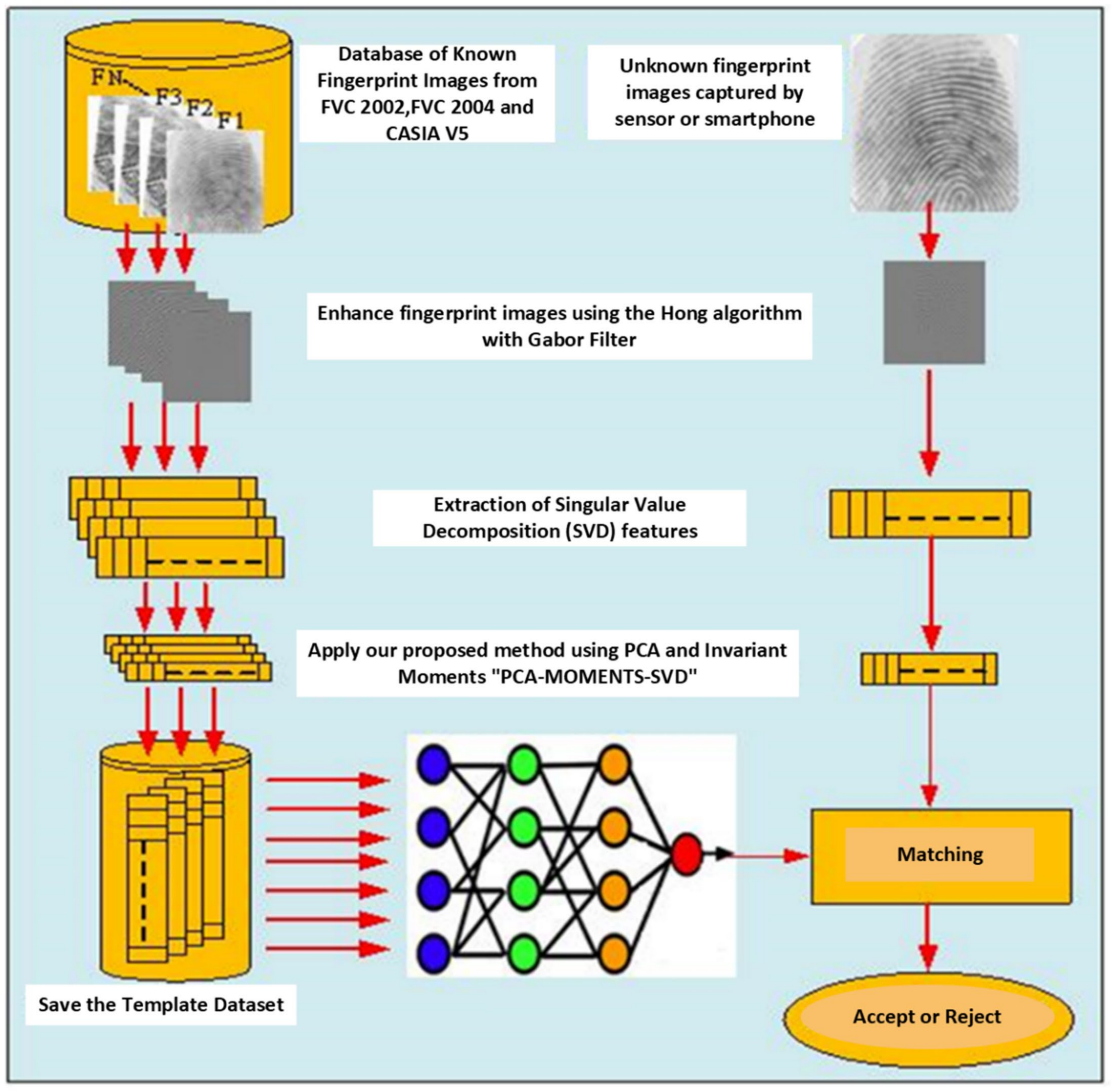
General process diagram for the proposed fingerprint recognition system (PCA-MOMENTS-SVD).

The proposed fully automatic matching approach relies on the fusion of seven invariant moments and SVD features for robust fingerprint identification. The process involves a neural network, as illustrated in Figure 1, outlining the fingerprint pattern identification and classification procedure. Verification between test and template fingerprint feature vectors is assessed using the absolute distance, the characterization degree and the Frobenuis norm as a similarity measure. This approach aims to overcome the limitations of traditional minutiae-based approaches by conducting extensive research, testing, and validating the effectiveness of modified Principal Component Analysis (PCA) with SVD and invariant moments.

## 2 Theoretical foundations of SVD and invariant moments

SVD is a powerful approach to matrix analysis that has applications beyond square matrices (Loperfido, 2015). This method breaks down a matrix into three fundamental building blocks: *U* and *V* are two unitary matrices, and *S* is a diagonal matrix. These resulting matrices capture the most important information from the original data. SVD ability to extract this inherent structure has made it a cornerstone in various signal and image processing tasks. The SVD decomposition is represented by Equation (1).


(1) $$A=\text{US}V^{t}$$

Invariant moments are unique image features that remain constant even when the image is scaled, rotated, or moved (Tiwari and Srivastava, 2024). This makes them extremely useful in image processing tasks. In our approach, we use moment analysis, see Equation (2), to extract these valuable features from the SVD feature vectors. This section delves more deeply into the concept of invariant moments.


(2) $$m_{pq}=\sum_{x=1}^{N}\sum_{y=1}^{N}x^{p}y^{q}f\left(x,y\right)$$

A moment of zero order corresponds to the object surface area and is expressed in the following form $m_{00}$ Equation (3):


(3) $$m_{00}=\sum_{x=1}^{N}\sum_{y=1}^{N}f\left(x,y\right)$$

The first-order moment is defined by the two expressions below (Equations 4 and 5):


(4) $$m_{10}=\sum_{x=1}^{N}\sum_{y=1}^{N}x\cdot f\left(x,y\right)$$

(5) $$m_{01}=\sum_{x=1}^{N}\sum_{y=1}^{N}y\cdot f\left(x,y\right)$$

They determine the centroid of the corresponding surface $\left(\bar{x},\bar{y}\right)$ (Equations 6 and 7):


(6) $$\bar{x}=\frac{m_{10}}{m_{00}}$$

(7) $$\bar{y}=\frac{m_{01}}{m_{00}}$$

The central moments of order (*p* and *q*) can be declared as Equation (8):


(8) $$\mu_{pq}=\sum_{x=1}^{N}\sum_{y=1}^{N}{\left(x-\bar{x}\right)}^{p}{\left(y-\bar{y}\right)}^{q}f\left(x,y\right)$$

The reduced and normalized central moments are defined as follows Equation (9):


(9) $$\eta_{pq}=\frac{\mu_{pq}}{{\mu^{\gamma }}_{00}}$$

with $\gamma =\frac{p+q}{2}+1$ for p+q=2,3,…

Several researchers have developed methods for extracting a specific set of seven features (Equations 10, 11), known as invariant moments, from an image (Hu, 1962; Yager and Amin, 2004; Liu et al., 2012; Monge-Alvarez et al., 2019; Hao et al., 2021). These features have a unique power: They remain constant even when the image is scaled, rotated, or moved around. This makes them extremely useful for tasks such as identifying patterns in images, which is exactly what our approach seeks to accomplish.


(10) $$\phi_{1}=\eta_{20}+\eta_{02}$$

(11) $$\phi_{2}={\left(\eta_{20}-\eta_{02}\right)}^{2}+4\eta^{2}$$

(12) $$\phi_{3}={\left(\eta_{30}-3\eta_{12}\right)}^{2}+{\left(3\eta_{21}-3\eta_{03}\right)}^{2}$$

(13) $$\phi_{4}={\left(\eta_{30}+\eta_{12}\right)}^{2}+{\left(\eta_{21}+\eta_{03}\right)}^{2}$$

(14) $$\begin{array}{c} \phi_{5}=\left(\eta_{30}-3\eta_{12}\right)\left(\eta_{30}+\eta_{12}\right)\left[{\left(\eta_{30}+\eta_{12}\right)}^{2}-3{\left(\eta_{21}+\eta_{03}\right)}^{2}\right]\\ +\left(3\eta_{21}-\eta_{03}\right)\left(\eta_{21}+\eta_{03}\right)\left[{\left(\eta_{30}+\eta_{12}\right)}^{2}-{\left(\eta_{21}+\eta_{03}\right)}^{2}\right] \end{array}$$

(15) $$\begin{array}{c} \phi_{6}=\left(\eta_{20}-\eta_{02}\right)\left(\eta_{30}+\eta_{12}\right)\left[{\left(\eta_{30}+\eta_{12}\right)}^{2}-{\left(\eta_{21}+\eta_{03}\right)}^{2}\right]\\ +4\eta_{11}\left(\eta_{30}+\eta_{12}\right)\left(\eta_{21}+\eta_{03}\right) \end{array}$$

(16) $$\begin{array}{c} \phi_{7}=\left(3\eta_{21}-\eta_{03}\right)\left(\eta_{30}+\eta_{12}\right)\left[{\left(\eta_{30}+\eta_{12}\right)}^{2}-3{\left(\eta_{21}+\eta_{03}\right)}^{2}\right]\\ +\left(3\eta_{12}-\eta_{30}\right)\left(\eta_{21}+\eta_{03}\right)\left[3{\left(\eta_{30}+\eta_{12}\right)}^{2}-{\left(\eta_{21}+\eta_{03}\right)}^{2}\right] \end{array}$$

## 3 Modified PCA with SVD and invariant moments

This section introduces a ground breaking feature extraction approach that is specifically designed to address the complexities of fingerprint verification. This method employs a powerful set of techniques, including SVD for data analysis, invariant moments for capturing key features that are resistant to variations such as rotation and scale, and neural networks for classification. A comprehensive flowchart (Figure 1) details the entire process.

In this case, known fingerprints are treated as feature vectors, with invariant moments providing valuable information. This combined approach, including our proposed modified PCA with SVD and invariant moments, leverages the strengths of each technique to achieve accurate fingerprint verification.

### 3.1 Input: enhanced fingerprint images

Hong et al. (1998) used the Gabor filter to improve fingerprint images. We use the same algorithm as Hong et al. to improve the input fingerprint images. First, we create the $F_{i}$ matrix, which contains all of the identified fingerprints. Indeed, i represents the reference within the fingerprint database, while m and n represent the dimensions of matrix $F_{i}$. Each image, assumed to have *M* pixels arranged in size *m* × *n*, is converted into a column vector $p_{i}$ of size *M* × 1. This vector contains the intensity values of all *M* pixels in a single column. Furthermore, set *S* containing *N* such fingerprint images can be represented as a matrix with dimensions *M* × *N*. Each column in this matrix corresponds to a single fingerprint vector ($p_{i}$).


(17) $$S=\left[p_{1},p_{2},p_{3},\ldots ,p_{N}\right]$$

The mean features $\bar{p}$ (Equation 18) of set *S* (Equation 17) are computed by taking the average of all $p_{i}$ vectors.


(18) $$\bar{p}=\frac{1}{N}\sum_{i=1}^{N}p_{i}$$

We arrange the data around zero by subtracting the average value $\bar{p}$ from each element in the original features. This creates a new matrix *T* (Equation 19) with the same dimensions (*M* × *N*).


(19) $$T=\left[{Ε}_{1},{Ε}_{2},{Ε}_{3},\ldots ,{Ε}_{N}\right]$$

(20) $${Ε}_{i}=p_{i}-\bar{p}$$

SVD of *T* is calculated, which results in singular values $\sigma_{i}$ and left and right singular vectors, $u_{i}$ and $v_{i}$, respectively. Matrix *T* (Equations 21 and 22) is represented as the sum of rank *r* components.


(21) $$T=\sigma_{1}u_{1}v_{1}^{t}+\sigma_{2}u_{2}v_{2}^{t}+\sigma_{3}u_{3}v_{3}^{t}+\ldots +\sigma_{r}u_{r}v_{r}^{t}$$

(22) $$T=\text{US}V^{t}=\sum_{i=1}^{r}\sigma_{i}u_{i}v_{i}^{t}$$

Calculating a scalar projection of ${Ε}_{i}$ allows us to determine how well each fingerprint $p_{i}$ matches the base images. This projection is represented by a set of values *u* assigned to each fingerprint. We build the general matrix $\Psi_{i}$ (Equations 23 and 24). This matrix is created by multiplying the transpose of the base fingerprint image vectors with ${Ε}_{i}$.


(23) $$\Psi_{i}={\left[u_{1},u_{2},\ldots ,u_{r}\right]}^{t}\left(p_{i}-\bar{p}\right)={\left[u_{1},u_{2},\ldots ,u_{r}\right]}^{t}\times {Ε}_{i}$$

(24) $$\Psi ={\left[u_{1},u_{2},\ldots ,u_{r}\right]}^{t}Ε$$

### 3.2 Feature extraction

For each $\Psi_{i}$ row, seven moment features $\left(\phi_{1},\phi_{2},\phi_{3},\phi_{4},\phi_{5},\phi_{6},\phi_{7}\right)$ are extracted and stored in the database. These attributes will be used as inputs for a neural network classifier.

## 4 Results and discussion

### 4.1 Fingerprint databases

Abdul Cader et al. (2023) carried out a thorough examination of fingerprint recognition systems in their work, emphasizing current developments and difficulties. They draw attention to how sensor technology and image capture affect the accuracy of the system. Their analysis examines how sensor modalities and underlying physical principles can introduce distortions during image capture. It covers contact and contactless (2D and 3D) fingerprint systems. In order to close this gap, Abdul Cader et al. (2023) offers a cutting-edge analysis that covers sensors, image acquisition, and interoperability issues with different fingerprint systems.

The FVC2002, FVC2004, FVC2006, and CASIA V5 fingerprint databases (Maio et al., 2000, 2002; Abdul Cader et al., 2023) provide a diverse set of fingerprint images for analysis, which we use in our experiment. Ten classes are available: four for FVC 2002 and 2004, one for FVC 2006, and one for CASIA V5. The following table provide a detailed description of the databases that were used in this work for the experimental study (Table 1).

**Table 1:** Characteristics of the FVC2002, FVC2004, FVC2006, and CASIA V5 fingerprint databases.

Database	Number of images	Number of people	Image size (pixels)	Image format
CASIA-fingerprint V5	20,000	500	328 × 356	BMP
FVC 2006	72,000	150	96 × 96	BMP
FVC 2002-DB1	880	110	388 × 374	BMP
FVC 2002-DB2	800	100	296 × 560	TIF
FVC 2002-DB3	1,440	180	300 × 300	BMP
FVC 2002-DB4	1,000	100	288 × 384	TIF
FVC 2004-DB1	1,100	100	640 × 480	TIF
FVC 2004-DB2	880	110	328 × 364	TIF
FVC 2004-DB3	1,440	120	300 × 480	TIF
FVC 2004-DB4	1,280	160	288 × 384	BMP

The FVC2004 and FVC2006 were built with temperature differential sensors, which use pyroelectric materials to convert temperature changes into voltage (Abdul Cader et al., 2023). In many fingerprint data acquisition systems, such as the FVC2004, the Atmel FingerChip is a standard component. The development of biometric technologies and the Internet of Biometric Things (IoBT) depends on these sensors. In addition to using a variety of sensors, the CASIA Fingerprint databases also make use of optical, ultrasonic, and capacitive sensors (Abdul Cader et al., 2023). Thermal sensors make up the majority of these sensors.

### 4.2 Discrimination performance using characterization degree and Frobenius norm

We choose 100 fingerprint images from each sub-database for analysis. Our goal is to determine whether enhanced fingerprint images improve the matching process over their original versions. To accomplish this, we propose a characterization degree, *J*, which will be computed for both sets of images (original and enhanced). This metric (*J*) is based on the ratio of variance between different fingerprints (inter-variance) to variance within a single fingerprint (intra-variance) (Amornraksa and Tachaphetpiboon, 2006; Balti et al., 2012, 2013).

A higher *J* value indicates a clearer distinction between different fingerprints, which can lead to better matching performance. For this evaluation, we use all 100 randomly selected fingerprint images from each sub-database, where ${\underline{x}}_{k,n}$ is the estimated feature vector for each fingerprint image (1 ≤ *k* ≤ 100 and 1 ≤ *n* ≤ 25).

The average of the $k^{th}$ fingerprint feature vector class is shown in Equation (25):


(25) $${\underline{m}}_{k}=\;\frac{1}{\;25}\;\sum_{n=1}^{25}{\underline{x}}_{k,n}$$

The mean of all features’ vector classes is also noted in Equation (26):


(26) $${\underline{m}}_{c}=\frac{1}{\;100}\;\sum_{k=1}^{100}{\underline{m}}_{k}$$

The matrix represents the mean of intra-class (within-class) dispersion matrices ${\underline{S}}_{\text{intra}}$ (Equation 27):


(27) $${\underline{S}}_{\text{intra}}=\frac{1}{\;2500}\;\sum_{k=1}^{100}\;\sum_{n=1}^{25}\;\left({\underline{x}}_{k,n}-{\underline{m}}_{k}\right)\;{\left({\underline{x}}_{k,n}-{\underline{m}}_{k}\right)}^{t}$$

This is the maximum likelihood estimate for the class covariance matrix. Additionally, the mean of between-class (inter-class) dispersion matrices describes the scattering of class sample means. The matrix computes this ${\underline{S}}_{\text{inter}}$ (Equation 28):


(28) $${\underline{S}}_{\text{inter}}=\frac{1}{40}\;\sum_{k=1}^{40}\;\left({\underline{m}}_{k}-{\underline{m}}_{c}\right)\;{\left({\underline{m}}_{k}-{\underline{m}}_{c}\right)}^{t}$$

Finally, the characterization degree (*J*) is calculated as (Equation 29):


(29) $$J=\text{trace}\;\left({S_{\text{intra}}}^{-1}\cdot S_{\text{inter}}\right)$$

This method extracts a feature vector from each ${\underline{x}}_{k,n}$ fingerprint image in the selected sets (original and enhanced). These feature vectors include both the mean and the variance, as well as higher-order moments up to the sixth order. The characterization degree (*J*), as previously described, is based on the ratio of inter-variance to intra-variance calculated from these features (Amornraksa and Tachaphetpiboon, 2006; Balti et al., 2012, 2013). A higher *J* value indicates a greater distinction between different fingerprints, which is desirable for a reliable fingerprint classification process. Table 2 compares the effectiveness of these features in achieving a high level of characterization.

**Table 2:** Characterization degrees (*J*) of examined features.

Characterization degree (*J*)	
Modified PCA with SVD and MOMENTS features (PCA-MOMENTS-SVD)	35.5
DCT_Features (Amornraksa and Tachaphetpiboon, 2006)	24.2
Invariant_Moment (Yang and Park, 2008)	23.3
Invariants and reduced features (Balti et al., 2013)	21.6

**Figure 2:**
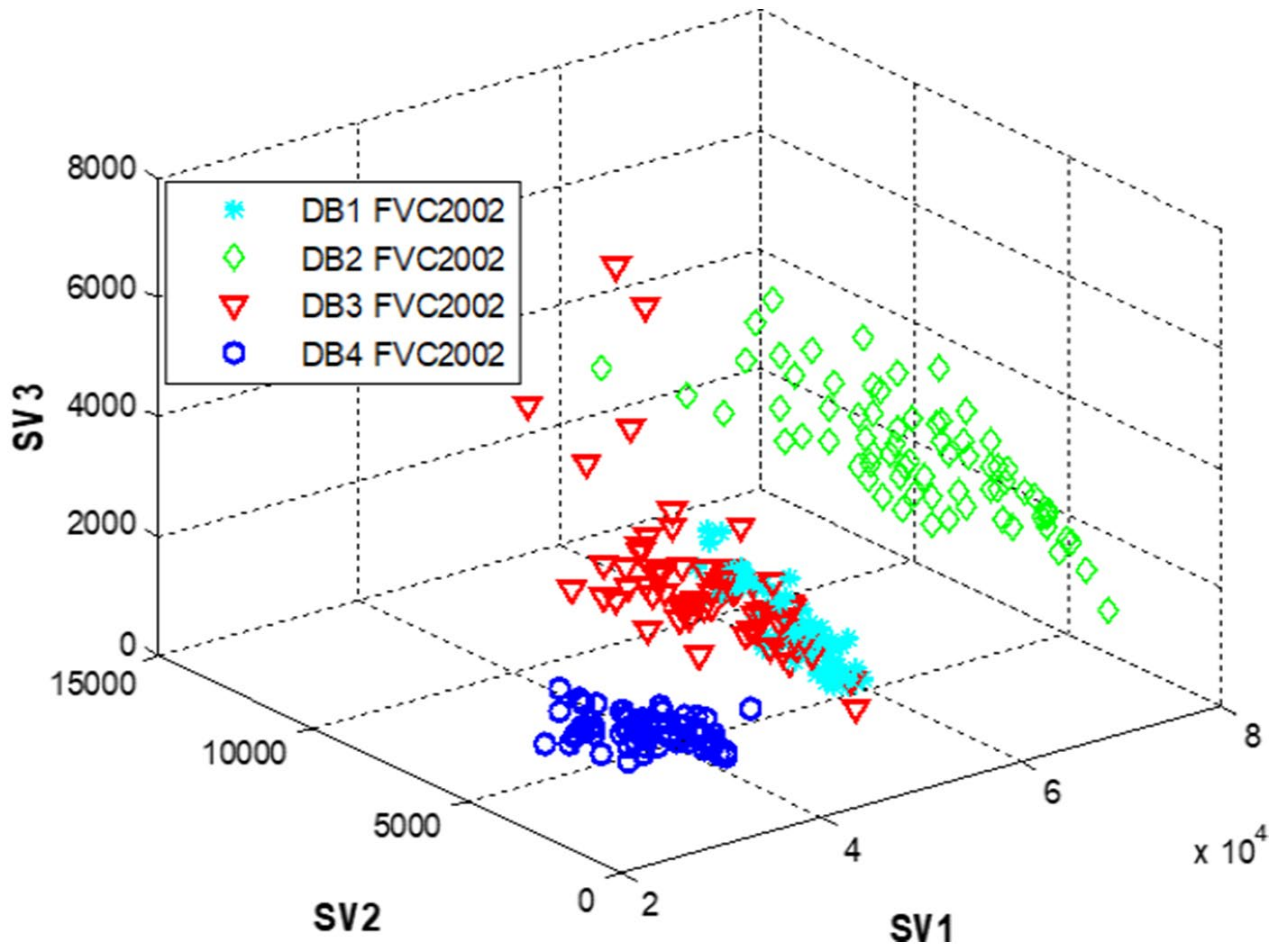
3D representation of singular fingerprint values.

Figure 2 shows the discriminative 3D of singular values obtained using the modified PCA with SVD and MOMENTS features approach.

To determine the potential improvement in fingerprint characterization achieved by our MOMENTS-SVD feature approach, we propose to evaluate the extracted SVD feature vectors using the Frobenius norm $\xi_{\text{Frobenius}}$ (Equation 33) for each fingerprint image. The Frobenius norm is a popular metric in image processing that provides a consistent measure of errors. In our context, a lower Frobenius norm for the SVD features may indicate a better representation of the fingerprint than the original data, potentially leading to enhanced characterization. Applying (Equations 30−32) to our experimental data.


(30) $$\|A\|{\;}_{F}=\sqrt{\sum_{i=1}^{m}\sum_{j=1}^{n}{\left|a_{ij}\right|}^{2}}=\text{trace}\;\left(A^{t}\;A\right)$$

The singular value can be used to calculate the Frobenius norm, where $\sigma_{i}$ represents the singular values of A.


(31) $$\|A\|{\;}_{F}=\sum_{i}{\sigma_{i}}^{2}$$

The suggested MOMENTS-SVD feature vectors are being evaluated. We generate the *E* projection vector, which is defined in equation (20) and apply it to the fingerprint images.


(32) $$F_{p}=\left[u_{1},u_{2},\ldots ,u_{r}\right]{\left[u_{1},u_{2},\ldots ,u_{r}\right]}^{t}Ε$$

(33) $$\xi_{\text{Frobenius}}=\frac{\|E-F_{p}\|{\;}_{F}}{\|E\|{\;}_{F}}$$

**Figure 3:**
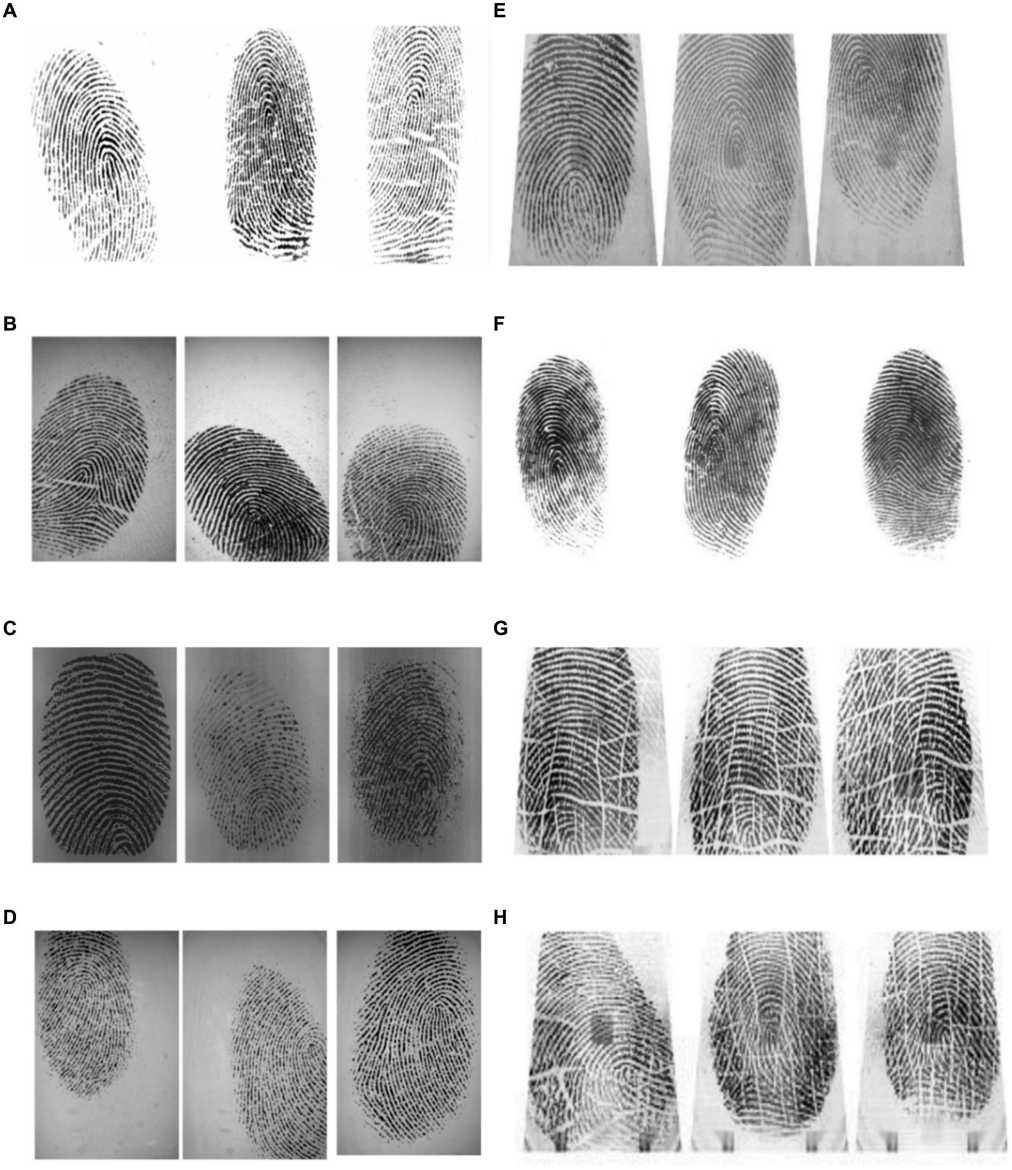
Examples from fingerprint databases commonly used in fingerprint recognition research: **(A)** FVC-2002 DB1, **(B)** FVC-2002 DB2, **(C)** FVC-2002 DB3, **(D)** FVC-2002 DB4, **(E)** FVC-2004 DB1, **(F)** FVC-2004 DB2, **(G)** CASIA-fingerprint V5, **(H)** Critical case images from CASIA-fingerprint V5.

To assess the effectiveness of SVD features for fingerprint characterization, we examine 25 grayscale fingerprints from the FVC2002 (Figure 3). SVD features are especially useful because they remain consistent even when fingerprints are rotated.

We conduct two controlled experiments to demonstrate the efficacy of our proposed SVD feature vectors in fingerprint matching.

**Figure 4:**
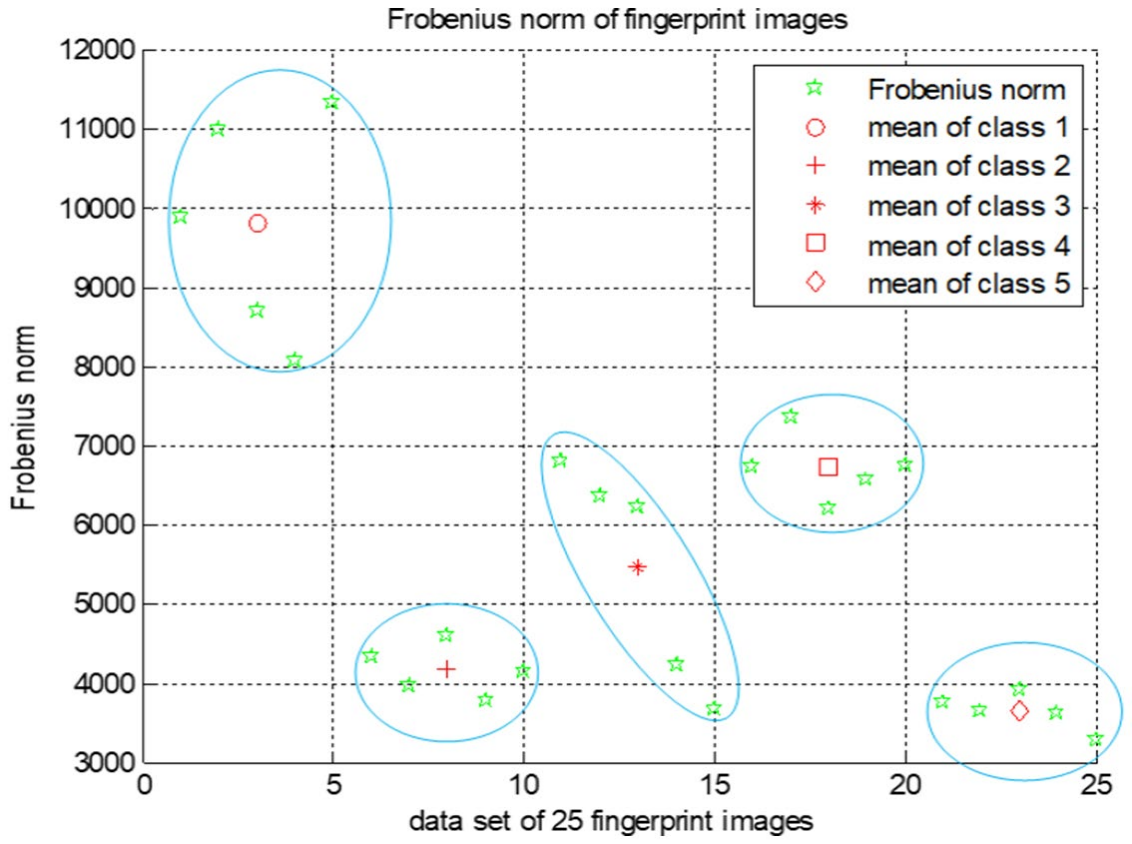
Frobenius norm for fingerprint images.

**Experiment 1: Fingerprint clustering (Figure** **4****):**

We choose a random set of fingerprints dataset (Figure 3). These fingerprints are then clustered using the proposed SVD feature extraction technique combined with the Frobenius norm. As shown in Figure 4, this approach effectively partitions fingerprints into distinct clusters (classes), demonstrating the SVD features ability to group similar fingerprints together.

**Experiment 2 Fingerprint matching accuracy:**

Our evaluation of the FVC fingerprint databases reveals a significant advantage of the proposed SVD-based representation for fingerprint indexing over the existing methods. The results highlight an important trade-off:

**Accuracy:** The suggested method with SVD feature matching provides superior verification accuracy.

**Speed:** Methods based on the Frobenius norm provide faster matching times, but at the potential cost of accuracy.

Furthermore, the proposed method has higher verification accuracy than other well-established techniques. These experiments show that SVD features outperform other techniques for matching fingerprints. They provide better verification system performance and greater resistance to variations in fingerprint image quality.

### 4.3 Performance evaluation

To assess the effectiveness of our fingerprint verification system, we use the standard protocol from the FVC2002, FVC2004, FVC2006, and CASIA V5 benchmark (Maio et al., 2000, 2002; Xie et al., 2020; Militello et al., 2021; Srivastava et al., 2022; Garg et al., 2024). This protocol is based on several key performance indicators:

**False Acceptance Rate (FAR):** This metric calculates the percentage of imposter fingerprints (those that do not match the claimed identity) which are mistakenly accepted as genuine. Having a lower FAR is desirable (Equation 34).

**False Reject Rate (FRR):** This metric measures the percentage of genuine fingerprints that the system incorrectly rejects. A lower FRR is also preferred (Equation 35).

**Equal Error Rate (EER):** This is the point at which FAR and FRR are equivalent. It serves as a common benchmark for comparing the performance of various fingerprint verification systems. A lower EER indicates improved overall performance.

**Genuine Acceptance Rate (GAR):** This metric measures the percentage of genuine fingerprints correctly accepted by the system. Having a higher GAR is desirable (Equation 36).

**NAG** (Number of Accepted Genuine Fingers): This metric measures the number of genuine fingerprints attempts correctly identified as belonging to authorized users. **TNG** (Total Number of Genuine Fingers): This metric measures the total number of attempts made with genuine fingerprints (authorized users). **NAI** (Number of Accepted Imposter Fingers): This metric measures the number of imposter fingerprint attempts incorrectly identified as belonging to authorized users (security failure).

**TNI** (Total Number of Imposter Fingers): This metric measures the total number of attempts made with imposter fingerprints (unauthorized users). **NRG** (Number of Rejected Genuine Fingers): This metric measures the number of genuine fingerprint attempts incorrectly rejected (causing user inconvenience).


(34) $$\text{FAR}=\frac{NAI}{TNI}$$

(35) $$FRR=\frac{NRG}{TNG}$$

(36) $$\text{GAR}=\frac{\text{NAG}}{TNG}$$

We calculate EER, FRR and FAR for all databases using matched genuine and impostor fingerprint pairs. For genuine matches, each test fingerprint is compared to the corresponding template from the same person. Imposter matches, on the other hand, involve comparing each test fingerprint to templates created by different people. The following section describes our proposed method’s identification performance.

We compare the performance of our suggested SVD feature-based approach to five well-known fingerprint verification techniques in order to assess its efficacy. This research uses five different approaches. The method proposed by Invariant Moment (Yang and Park, 2008) combines BPNN and invariant moment features. Discrete Cosine Transform (DCT) features were utilized in the Second Method, DCT Features, developed by Amornraksa and Tachaphetpiboon (2006), to match fingerprints. The Third Method, Neuro Fuzzy method, was proposed by Srivastava et al. (2022), the Fourth Method, ResNet method, was proposed by Militello et al. (2021), and the Fifth Method, VGG19, was proposed by Garg et al. (2024).

The five methods were put into practice. In the framework of these well-known techniques, our experiment compares two matching strategies: absolute distance and neural network (Tables 3, 4).

**Table 3:** Performance comparison of proposed method with the absolute distance (D_abs) for different databases and methods (EER %).

Database	Invariant moment Yang and Park (2008)	DCT features Amornraksa and Tachaphetpiboon (2006)	Proposed method: MOMENTS-SVD
FVC 2002	4.8	6.3	2.1
FVC 2004	4.1	6.7	2.7
FVC 2006	4.0	6.2	2.3
CASIA V5	3.8	5.4	2.2

**Table 4:** Comparison of EER (%) with NN classifier for different databases and methods.

Database	Invariant moment Yang and Park (2008)	DCT features Amornraksa and Tachaphetpiboon (2006)	Neuro fuzzy Srivastava et al. (2022)	ResNet Militello et al. (2021)	VGG19 Garg et al. (2024)	Proposed method
FVC 2002	0.9	0.89	0.87	0.88	0.9	0.82
FVC 2004	0.88	0.86	0.84	0.71	0.7	0.69
FVC 2006	0.86	0.85	0.83	0.68	0.65	0.55
CASIA V5	0.75	0.73	0.72	0.65	0.61	0.49

### 4.4 Role of absolute distance in fingerprint recognition

Absolute distance, also known as Euclidean distance, is used in fingerprint recognition systems to quantify the similarity between feature vectors extracted from captured fingerprints and the corresponding feature vectors of template fingerprints stored in a database.

Vectors $\phi_{i}$ refers to the invariant feature extracted from the input fingerprint, while ${\phi_{i}}^{t}$ is the feature vector obtained from the database for the template fingerprint. Added to that, $V_{d}$ (Equation 37) represents the difference vector computed from their respective feature vectors.


(37) $$V_{d}=\;\left(\frac{\left|\phi_{1}^{t}-\phi_{1}\right|}{\max\;\left(\phi_{1}^{t},\phi_{1}\right)},\frac{\left|\phi_{2}^{t}-\phi_{2}\right|}{\max\;\left(\;\phi_{2}^{t},\phi_{2}\right)},\ldots \frac{\left|\phi_{7}^{t}-\phi_{7}\right|}{\max\;\left(\;\phi_{7}^{t},\phi_{7}\right)}\right)$$

The absolute distance $D_{\text{abs}}$ (Equation 38) between the two invariant feature vectors is defined as:


(38) $$D_{\text{abs}}=\sum_{i=1}^{7}\;\frac{\left|\;\phi_{i}^{t}-\phi_{i}\right|}{\max\;\left(\;\phi_{i}^{t},\phi_{i}\;\right)}$$

Table 3 compares the Equal Error Rate (EER) attained by various techniques using a variety of fingerprint verification databases, with an emphasis on the Absolute Distance (D_abs) metric. The proposed method, MOMENTS-SVD, is compared with Invariant Moment (Yang and Park, 2008), DCT Features (Amornraksa and Tachaphetpiboon, 2006), and the databases evaluated are FVC 2002, FVC 2004, FVC 2006, and CASIA V5. With values of 2.1, 2.7, 2.3, and 2.2% for FVC 2002, FVC 2004, FVC 2006, and CASIA V5, respectively. The results show that the suggested method consistently achieves the lowest EER across for all databases.

Compared to the Invariant Moment and DCT Features methods, which have higher EER values ranging from 3.8 to 6.7%, this performance is noticeably better.

## 5 Neural networks for fingerprint classification

**Figure 5:**
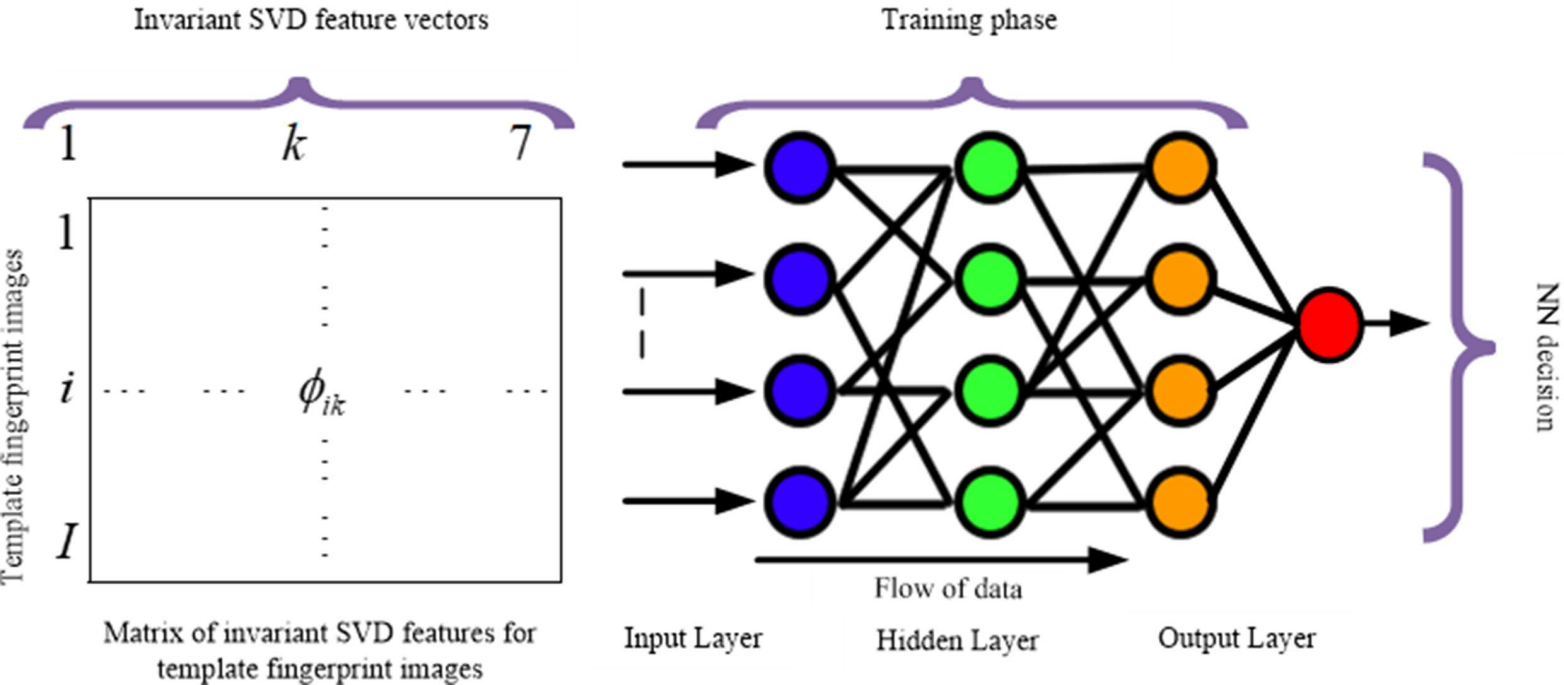
Simplified neural network architecture for fingerprint identification.

This study expands on previous research in fingerprint verification with supervised neural networks (Yang and Park, 2008; Balti et al., 2013; Garg et al., 2024). However, our primary focus is on fingerprint identification, which entails categorizing fingerprints rather than determining whether they match a specific template. The neural network architecture has three layers (Figure 5): input, hidden, and output. For nonlinearity, we use sigmoid activation functions in the hidden layer, while for classification we use linear activation functions in the output layer (Figure 5).

After 893 epochs of experimentation, we find the optimal configuration with 60 hidden neurons and a mean squared error target of 10^−20^.

A comparative study of the Equal Error Rate (EER) attained by various feature extraction and classification techniques across multiple fingerprint verification databases is shown in Table 4. The databases that were examined are CASIA V5, FVC 2002, FVC 2004, and FVC 2006. The approaches that are compared are the following: ResNet (Militello et al., 2021), VGG19 (Garg et al., 2024), Neuro Fuzzy (Srivastava et al., 2022), DCT Features (Amornraksa and Tachaphetpiboon, 2006), and Invariant Moment (Yang and Park, 2008). The findings show that, for all databases, the suggested method consistently produces the lowest EER, with values for FVC 2002, FVC 2004, FVC 2006, and CASIA V5 of 0.82, 0.69, 0.55, and 0.49%, respectively.

This performance outperforms the other approaches, indicating the superior accuracy and robustness of the suggested method in fingerprint verification. The EER of the other methods, which highlight the consistent enhancement offered by the suggested approach, range from 0.87 to 0.9% for FVC 2002, 0.7–0.88% for FVC 2004, 0.65–0.86% for FVC 2006, and 0.61–0.75% for CASIA V5.

**Figure 6:**
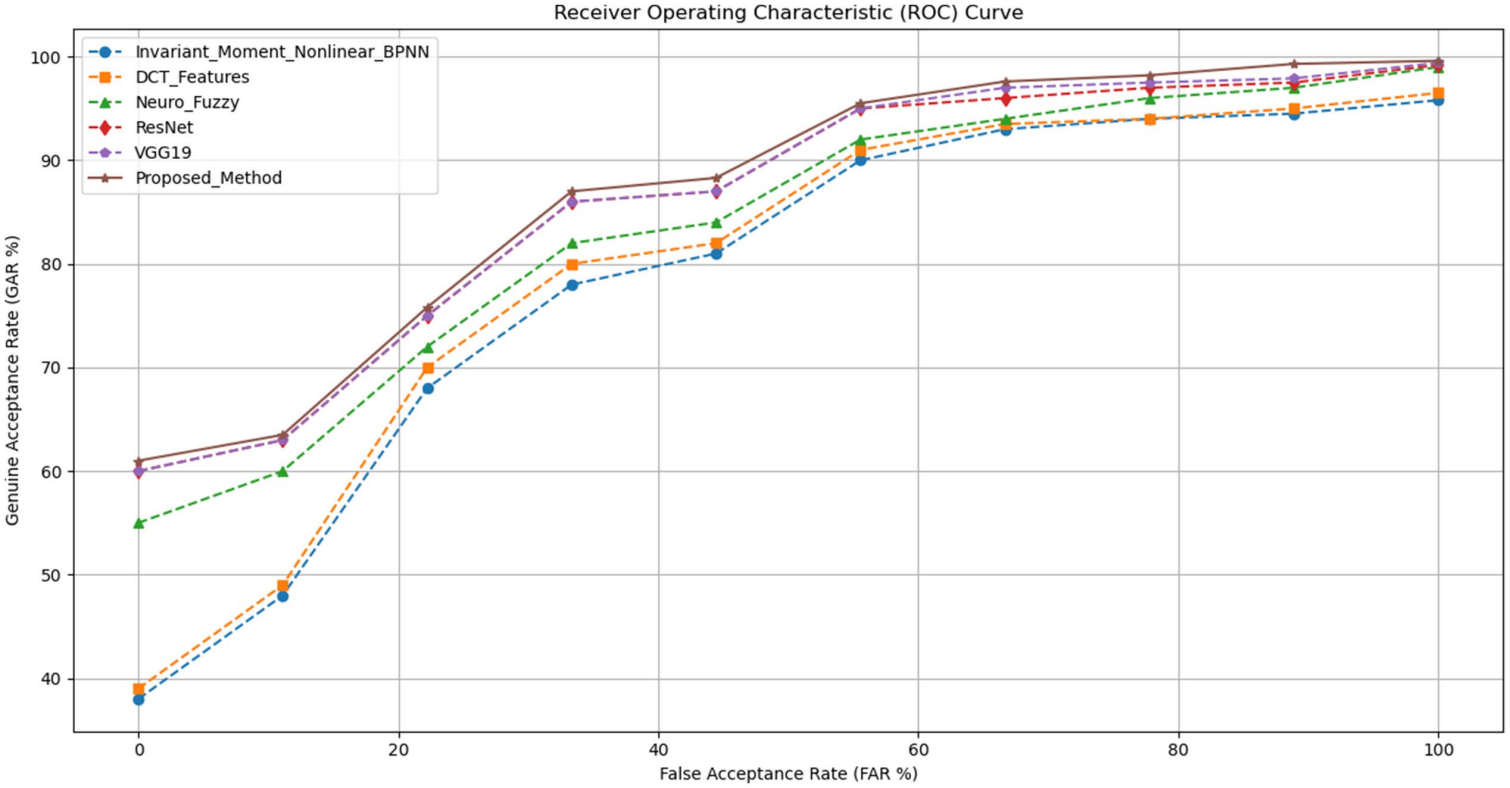
ROC analysis.

We also use the Receiver Operating Characteristic (ROC) curve (Figure 6), which compares the GAR to the FAR%, to assess these methods more effectively. Through graphical demonstration of maintaining a higher GAR for a lower FAR%, this analysis validates the superiority of the proposed method in biometric authentication technology. In order to improve security and user confidence in authentication procedures, these results demonstrate the usefulness of ROC analysis and GAR percentages in evaluating the precision and dependability of biometric recognition systems. A thorough assessment of the efficacy of various biometric recognition methods is made possible by the True Acceptance Rate (GAR) percentages for varying False Acceptance Rates (FAR). With GAR percentages ranging from 61 to 99.6%, the suggested method performs better than every other method that was tested. This performance improvement demonstrates the extent to which the proposed method can accurately identify real users at different security levels.

## 6 Conclusion

In conclusion, this paper presents a new fingerprint verification system that improves identification accuracy by utilizing SVD features and invariant moments. The matching engine of the system is a neural network that extracts and compares these features from captured fingerprints (tests) and database-stored templates. This method performs better in terms of flexibility, robustness against sensor noise, and achieving high matching accuracy when compared to conventional metrics like absolute distance and Frobenius norm. Neural network integration holds great promise for improving biometric authentication systems and providing a dependable solution for safe and effective identification procedures in a variety of applications. In the future, studies could focus on improving neural network architectures and diversifying datasets to improve the system’s functionality and applicability in real scenarios.

## Data availability statement

The raw data supporting the conclusions of this article will be made available by the authors, without undue reservation.

## Author contributions

AB: Conceptualization, Data curation, Formal analysis, Investigation, Methodology, Software, Supervision, Validation, Visualization, Writing – original draft, Writing – review & editing. AH: Conceptualization, Formal analysis, Methodology, Software, Validation, Writing – original draft, Writing – review & editing. SA: Formal analysis, Methodology, Supervision, Validation, Writing – review & editing. MB: Formal analysis, Methodology, Software, Supervision, Validation, Writing – review & editing. MS: Project administration, Supervision, Writing – review & editing.
